# Octreotide for the Management of Gastrointestinal Bleeding in a Patient with a HeartWare Left Ventricular Assist Device

**DOI:** 10.1155/2014/826453

**Published:** 2014-12-18

**Authors:** Geetanjali Dang, Ryan Grayburn, Geoffrey Lamb, Adrian Umpierrez De Reguero, Nunzio Gaglianello

**Affiliations:** ^1^Department of Medicine, Medical College of Wisconsin, Milwaukee, WI 53226, USA; ^2^Advanced Heart Failure and Transplant Cardiology, Medical College of Wisconsin, Milwaukee, WI 53226, USA

## Abstract

HeartWare is a third generation left ventricular assist device (LVAD), widely used for the management of advanced heart failure patients. These devices are frequently associated with a significant risk of gastrointestinal (GI) bleeding. The data for the management of patients with LVAD presenting with GI bleeding is limited. We describe a 56-year-old lady, recipient of a HeartWare device, who experienced recurrent GI bleeding and was successfully managed with subcutaneous (SC) formulations of octreotide.

## 1. Case Report

The patient is a 56-year-old woman with nonischemic cardiomyopathy who was implanted with a HeartWare left ventricular assist device (LVAD) as a bridge to transplantation (BTT) and had an uneventful postoperative course. The initial anticoagulation regimen consisted of a vitamin K antagonist (warfarin) with a goal international normalized ratio (INR) of 2-3, aspirin of 324 mg daily, and dipyridamole of 125 mg three times daily. She received intravenous heparin until she was bridged to a goal INR of 2-3, following which the heparin infusion was stopped.

Five weeks following discharge, the patient was evaluated in the clinic for complaints of dizziness and light-headedness with postural variations. She denied any abdominal pain, nausea, vomiting, melena, hematochezia, or bleeding from any other sites. Lab work revealed hemoglobin (Hb) of 3.7, hematocrit (Hct) of 13, and an INR of 2.7, necessitating blood transfusions. Fecal occult blood was positive. An esophagogastroduodenoscopy (EGD), colonoscopy, and capsule endoscopy were performed which did not reveal any active source of bleeding. The dose of aspirin was reduced to 81 mg daily; dipyridamole was stopped while warfarin was continued with the goal INR of 2-3. Her LVAD pump speed was also reduced from 2600 rpm to 2400 rpm in an effort to achieve increased pulsatility, assessed by echocardiogram to ensure her aortic valve opened with each cardiac cycle. She had several repeat hospital admissions with similar complaints, each requiring multiple blood transfusions every week despite stopping the aspirin and reducing the goal INR to 1.8–2.5. Three weeks later, she was admitted with suction alarms from the HeartWare and was found to have Hb of 6.6 requiring further transfusion. Repeat EGD and capsule endoscopy were negative. By this time, she had received 30 units of packed red blood cells. She was started on 100 *μ*g SC octreotide twice daily. Over a period of three days, her Hb remained stable at 8.6 without further transfusions. Octreotide therapy was then held due to its lack of evidence in this setting and a theoretical increased risk of platelet activation. The following day, hemoglobin trended down to 7.1. Another blood transfusion was given, bringing her Hb back up to 9.4, and she was restarted on 100 *μ*g of SC octreotide twice daily. Her Hb remained stable at 9.4 for 24 hours and she was discharged home on twice daily doses of SC octreotide of 100 *μ*g. She did not require any transfusions for 3 weeks, following which she was once again admitted with low Hb and needed further transfusions. Repeat EGD was performed and showed one actively oozing erosion in the body of the stomach, two nonbleeding arteriovenous malformations in the distal duodenum, and two actively oozing arteriovenous malformations in the proximal jejunum all of which were ablated. Her Hb stabilized prior to discharge. She was continued on 100 *μ*g of SC octreotide twice daily in the hospital and then switched to 20 mg IM injections every month for ease of administration. Her Hb remained stable at 10 g/dL over the subsequent 8 weeks.

## 2. Discussion and Review of the Literature

Left ventricular assist devices (LVADs) are frequently used for the management of advanced heart failure patients [1]. Continuous flow LVADs are preferred over the pulsatile flow LVADs due to smaller size and less surgical trauma; however, they pose an increased risk for gastrointestinal (GI) bleeding [2]. Several theories have been proposed as the possible mechanism of bleeding in these patients, including reduced arterial pressure leading to GI arteriovenous malformations, development of Von Willebrand syndrome, and mucosal ischemia [3]. There are no clear recommendations for the management of patients with LVAD presenting with GI bleeding. Here, we describe a patient with HeartWare LVAD implant who presented with recurrent episodes of GI bleeding. She required weekly blood transfusions despite multiple GI investigations, stopping antiplatelet medications and lowering her INR goal, and efforts to maintain pulsatility by reducing her LVAD speed. However, her Hb finally stabilized after starting octreotide therapy.

Prior studies have shown that the use of long acting somatostatin analogues in patients with angiodysplasia has reduced the frequency of recurrent bleeding and need for further transfusion, though not completely eliminating it [4]. There is very limited data about the use of octreotide in LVAD patients presenting with GI bleeding. In 2013, Rennyson et al. described a patient with HeartMate II presenting with recurrent GI bleeding who was initially managed with 100 *μ*g SC octreotide and then was discharged home on intramuscular (IM) 20 mg Depot of octreotide. He responded with decreased frequency of GI bleeding and reduced need for subsequent transfusions [5]. In 2014, Coutance et al. described another patient with Jarvik 2000 LVAD as destination therapy presenting with recurrent episodes of GI bleeding, who was effectively managed with long acting monthly dose of IM octreotide over a 23-month follow-up period [6]. In 2012, a small single institution study demonstrated that using octreotide for management of GI bleeding in patients with LVADs did not result in any significant difference in the length of stay, units of packed red blood cells administered, rebleeding episodes, or mortality [7]. Another single institute experience described successful use of octreotide infusion (25 *μ*g/min), SC octreotide (100 *μ*g twice daily), or intramuscular injections of octreotide (10 mg monthly) for the management of 5 cases of GI bleeding occurring in patients with rotatory LVADs [8].

The above data suggests that octreotide may be a viable option for LVAD patients with recurrent episodes of GI bleeding. This therapy is especially attractive for BTT patients in whom recurrent blood transfusions are avoided due to possible sensitization. However, further studies are required to validate these findings in larger patient population and comparison of its use in pulsatile versus continuous flow devices before this can become a standard of care in such patients.

## Supplementary Material

The above picture is a graphical representation of the Hemoglobin (Hb) trend of our patient over a 5 month period. It has several troughs and peaks representing the Hb drop and blood transfusion respectively until the octreotide was started as indicated by the black arrow. Following the initiation of octreotide, The Hb has remained steady above 9 gm over 8 week period of follow up.

## References

[1] Hunt S. A., Abraham W. T., Chin M. H. (2005). ACC/AHA 2005 guideline update for the diagnosis and management of chronic heart failure in the adult: a report of the American College of Cardiology/American Heart Association Task Force on Practice Guidelines. *Circulation*.

[2] Loor G., Gonzalez-Stawinski G. (2012). Pulsatile vs. continuous flow in ventricular assist device therapy. *Best Practice and Research: Clinical Anaesthesiology*.

[3] John R., Lee S. (2009). The biological basis of thrombosis and bleeding in patients with ventricular assist devices. *Journal of Cardiovascular Translational Research*.

[4] Bon C., Aparicio T., Vincent M., Mavros M., Bejou B., Raynaud J.-J., Zampeli E., Airinei G., Sautereau D., Benamouzig R., Michopoulos S. (2012). Long-acting somatostatin analogues decrease blood transfusion requirements in patients with refractory gastrointestinal bleeding associated with angiodysplasia. *Alimentary Pharmacology & Therapeutics*.

[5] Rennyson S. L., Shah K. B., Tang D. G., Kasirajan V., Pedram S., Cahoon W., Malhotra R. (2013). Octreotide for left ventricular assist device-related gastrointestinal hemorrhage: can we stop the bleeding?. *ASAIO Journal*.

[6] Coutance G., Saplacan V., Belin A., Repessé Y., Buklas D., Massetti M. (2014). Octreotide for recurrent intestinal bleeding due to ventricular assist device. *Asian Cardiovascular and Thoracic Annals*.

[7] Aggarwal A., Pant R., Kumar S., Sharma P., Gallagher C., Tatooles A. J., Pappas P. S., Bhat G. (2012). Incidence and management of gastrointestinal bleeding with continuous flow assist devices. *Annals of Thoracic Surgery*.

[8] Hayes H. M., Dembo L. G., Larbalestier R., O'Driscoll G. (2010). Management options to treat gastrointestinal bleeding in patients supported on rotary left ventricular assist devices: a single-center experience. *Artificial Organs*.

