# Supplementary material for: Octreotide for the Management of Gastrointestinal Bleeding in a Patient with a HeartWare Left Ventricular Assist Device
Source: Case Rep Cardiol. 2014 Dec 18;2014:826453. doi: 10.1155/2014/826453 (PMC4281453; doi:10.1155/2014/826453)
Supplement: Supplementary file 1 — The above picture is a graphical representation of the Hemoglobin (Hb) trend of our patient over a 5 month period. It has several troughs and peaks representing the Hb drop and blood transfusion respectively until the octreotide was started as indicated by the black arrow. Following the initiation of octreotide, The Hb has remained steady above 9 gm over 8 week period of follow up. [file 826453.f1.docx]

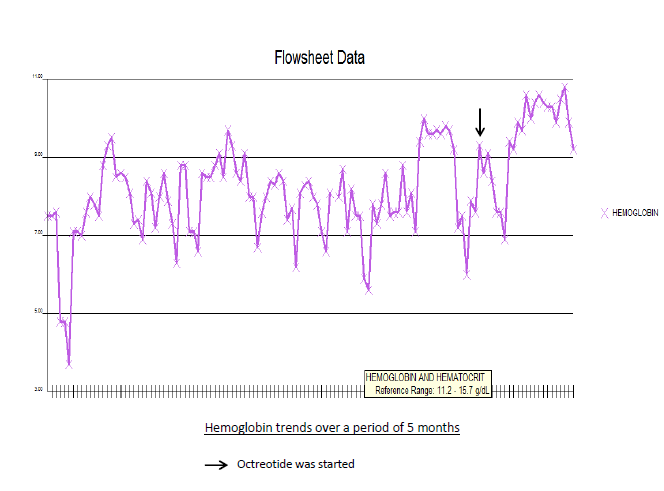


The above picture is a graphical representation of the Hemoglobin (Hb) trend of our patient over a 5 month period. It has several troughs and peaks representing the Hb drop and blood transfusion respectively until the octreotide was started as indicated by the black arrow. Following the initiation of octreotide, The Hb has remained steady above 9 gm over 8 week period of follow up.
